# A porcine polytrauma model with two different degrees of hemorrhagic shock: outcome related to trauma within the first 48 h

**DOI:** 10.1186/s40001-015-0162-0

**Published:** 2015-09-04

**Authors:** D. Eschbach, T. Steinfeldt, F. Hildebrand, M. Frink, K. Schöller, M. Sassen, T. Wiesmann, F. Debus, N. Vogt, E. Uhl, H. Wulf, S. Ruchholtz, H. C. Pape, K. Horst

**Affiliations:** Center for Orthopaedics and Trauma Surgery; University Hospital Giessen and Marburg, Marburg, Germany; Department of Anaesthesiology and Critical Care, University of Marburg, Marburg, Germany; Department of Neurosurgery, University of Giessen, Giessen, Germany; Trauma Department, University of Aachen, Aachen, Germany

**Keywords:** Experimental animal model, Porcine polytrauma model, Combined trauma model

## Abstract

**Background:**

An animal polytrauma model was developed, including trunk and extremity injuries combined with hemorrhagic shock and a prolonged post-traumatic phase. This could be useful for the assessment of different therapeutic approaches during intensive care therapy.

**Methods:**

A standardized polytrauma including lung contusion, liver laceration and lower leg fracture was applied in 25 pigs. They underwent controlled haemorrhage either with a blood volume loss of 45 % and a median arterial pressure (MAP) <30 mmHg/90 min (group L, *n* = 15) or a 50 % blood loss of and an MAP <25 mmHg/120 min (group H, *n* = 10). Five non-traumatized pigs served as a control (group C). Subsequently, intensive care treatment was given for an observational period of 48 h.

**Results:**

Both trauma groups showed signs of shock and organ injury (heart rate, MAP and lactate). The frequency of cardiopulmonary resuscitation (CPR) and lung injury was directly related to the severity of the haemorrhagic shock (CPR—group L: 4 of 15 pigs, group H: 4 of 10 pigs; Respiratory failure—group L: 3 of 13, group H: 3 of 9. There was no difference in mortality between trauma groups.

**Conclusion:**

The present data suggest that our model reflects the mortality and organ failure of polytrauma in humans during shock and the intensive care period. This suggests that the experimental protocol could be useful for the assessment of therapeutic approaches during the post-traumatic period.

## Background

There are several animal models for trauma and organ failure research, but a model mimicking real polytrauma, including simultaneous organ and musculoskeletal injuries, has not yet been described. Recently we described an animal experimental model of multi-system injury, consisting of blunt chest trauma, penetrating abdominal trauma and pressure-controlled hemorrhagic shock in pigs with an observational period of 16 h [1]. This model lacked a musculoskeletal injury component, and the study period was short by clinical standards. Most models lack information on the latter, i.e., 15-h-plus, post-traumatic phase [2]. Indeed, most studies of new early phase treatments do not adequately reflect the clinical situation in humans.

The aim of the present experiment was to establish a porcine model of hemorrhagic shock, multi-organ injury and musculoskeletal trauma with a prolonged observational period of 48 h. To standardize treatment during the post-trauma period, management of temperature, cardiovascular complications, volume therapy, and the prevention and treatment of ventilator or trauma-associated lung complications were defined prior to the study. Moreover, we compared two different severities of hemorrhagic shock to identify the most appropriate trauma intensity with respect to the occurrence of trauma-related organ failure.

## Methods

### Structural and financial investigation requirements

The experimental setup was time consuming and resource intensive. The experiment took place 5 days a week for 24 h a day, over a period of 6 months. Seven physicians, one veterinarian, four students, one medical technician and one nurse anesthetist were trained for 6 weeks during the feasibility study. The animals were handled and fed by four members of the animal care team. We implemented an operational shift system, with six people present from 7:00 a.m. to 7:00 p.m. 3 days a week (Monday, Wednesday and Friday). This was for assessment of new animals or performance of euthanasia and standardized section. Three team members were present 2 days a week (Tuesday and Thursday) from 7:00 a.m. to 7:00 p.m., while two people were responsible for the night shift (7:00 p.m. to 7:00 a.m.). The night shift team included an experienced physician. The total study expenditure for animals and material resources amounted to approximately €55,000. Personal effort was borne by members of the four participating institutions.

### Animals

The experimental procedures were approved by local authorities (Ref. 22/2013) Regional board, Giessen, Germany and the study was performed in compliance with the Helsinki convention for the use and care of animals and reported in consent with the ARRIVE guidelines [3]. In this study, 30 male pigs (Deutsche Landrasse) weighing 29.7–41.5 kg (median 34.6 kg) were used. All pigs were male, aged 3–6 months, healthy and fasted for 12 h at the start of investigation. Pigs were delivered twice a week from a nearby animal breeding service.

### Experimental groups

Five non-traumatized pigs served as controls (group C). Standardized polytrauma with a “low” blood loss was induced in 15 pigs (group L). In a second polytrauma group a “high” blood loss was induced and was applied in 10 pigs (group H). The target median arterial pressure was to be maintained at least 5 mmHg lower in group H by escalated hemorrhage and shock period was extended from 60 to 90 min. Animals were randomly assigned to intervention or control groups.

### Instrumentation and measurements

The animals were premedicated with diazepam (1 mg/kg), ketamine (20 mg/kg) and atropine (0.5 mg) intramuscularly. They were placed in the prone position and pre-oxygenated (10 LO_2_/min). After ear vein cannulation, anesthesia was induced with sufentanil and disoprivane followed by a continuous infusion (sufentanil 0.8 µg/kg/h, disoprivane 3–4 mg kg/h) during the entire study period of 48 h. The animals were orotracheally intubated and given pressure-controlled ventilation with a tidal volume of 6–8 mL kg^−1^ (Draeger, Evita, Danvers, MA, USA), positive end-expiratory pressure (PEEP) of 5 cm H_2_O, FiO_2_ of 0.3, and inspiration-to-expiration ratio (I:E) of 1:2. Respiratory rate was varied to achieve an endtidal CO_2_ less than 7.3 kPa. Ventilatory assessment was performed under control of blood gas analysis (BGA, ABL 700, Radiometer Copenhagen). A balanced saline solution (Ringer’s acetate) was continuously infused at a rate of 2 mL/kg/h. Hypovolemia was treated with a fluid bolus of 10 mL/kg. A single shot of cefuroxim 80 mg/kg was applied prior to the interventions.

The following catheters were inserted under aseptic conditions: an arterial PiCCO system (pulse contour cardiac output) in the left femoral artery, a central venous line via the right jugular vein (3 lumen HD, Arrow, PA, USA), a suprapubic urine catheter, and a two-lumen hemodialysis line was placed in the left femoral vein (two-lumen HD, 14Fr, 15 cm, Arrow, PA, USA). Tracheotomy was then performed. All ventilator and hemodynamic parameters were monitored and recorded. Electrocardiogram, oxygen saturation, temperature and arterial pressure were monitored continuously.

### Experimental protocol

**Fig. 1:**
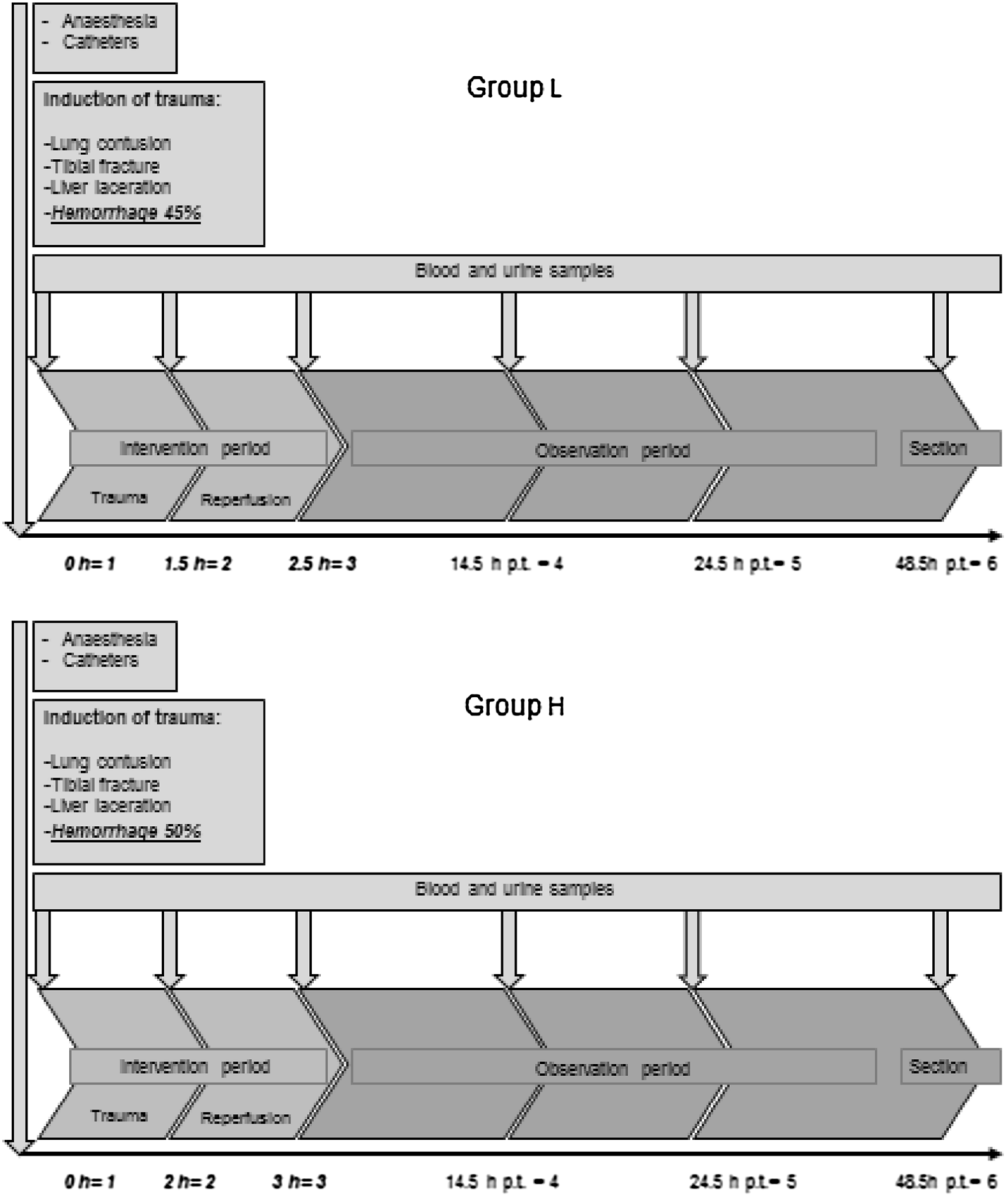
Time flow in experimental setup of groups L and H. Points of analysis of blood and urine samples are marked and differences in setup pointed out

**Fig. 2:**
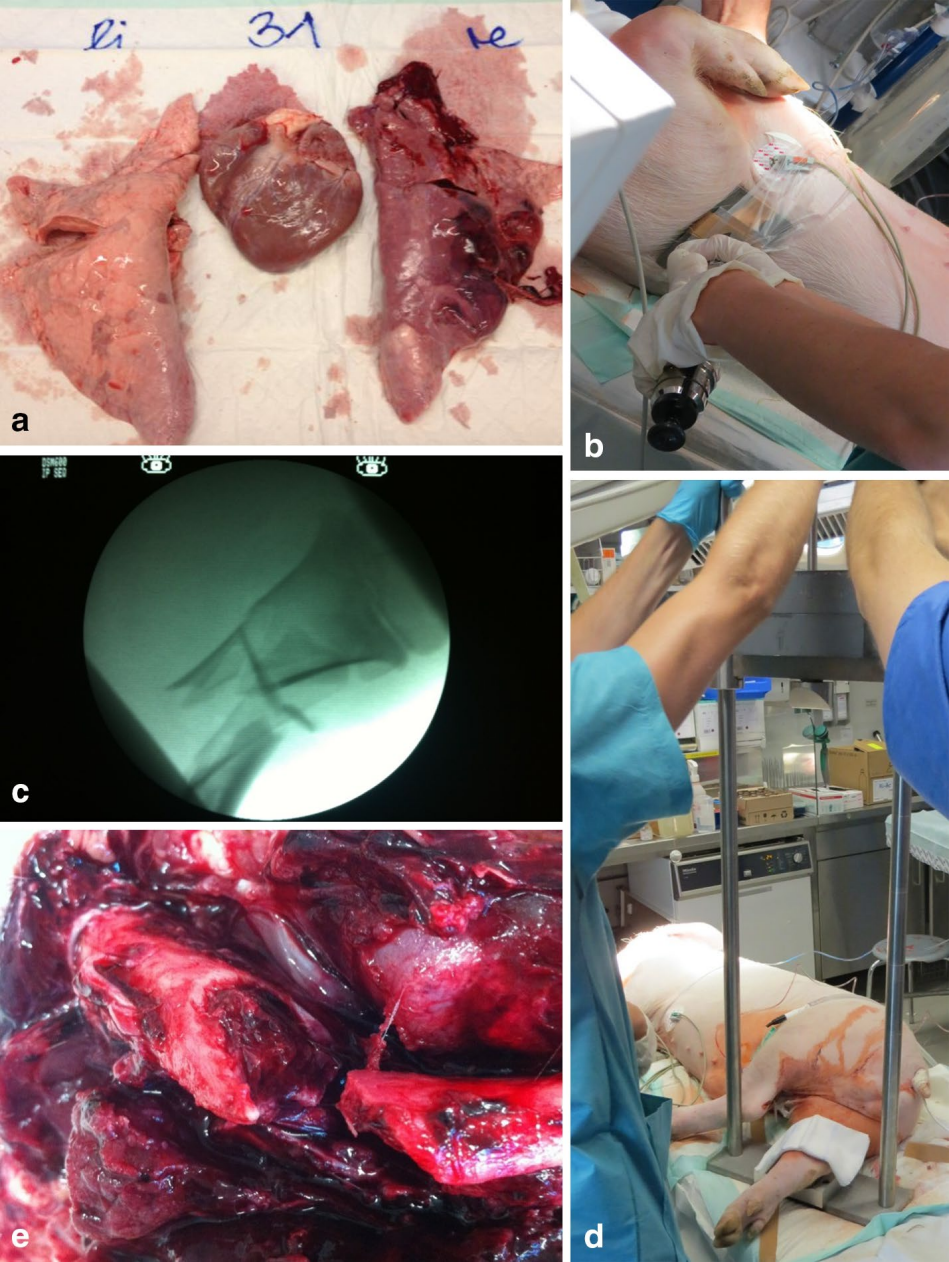
Autopsy findings and experimental setup. **a** Right lung shows macroscopic tissue damage and hemorrhagic bullae. **b** Assessment during the performance of lung trauma. The captive bolt stunner is targeted towards the lead plate. **c** Fluoroscopy of right hind leg after fracture. **d** Drop-weight gadgetry placed above the right hind leg to produce fracture. **e** Macroscopic findings in the fracture zone

**Fig. 3:**
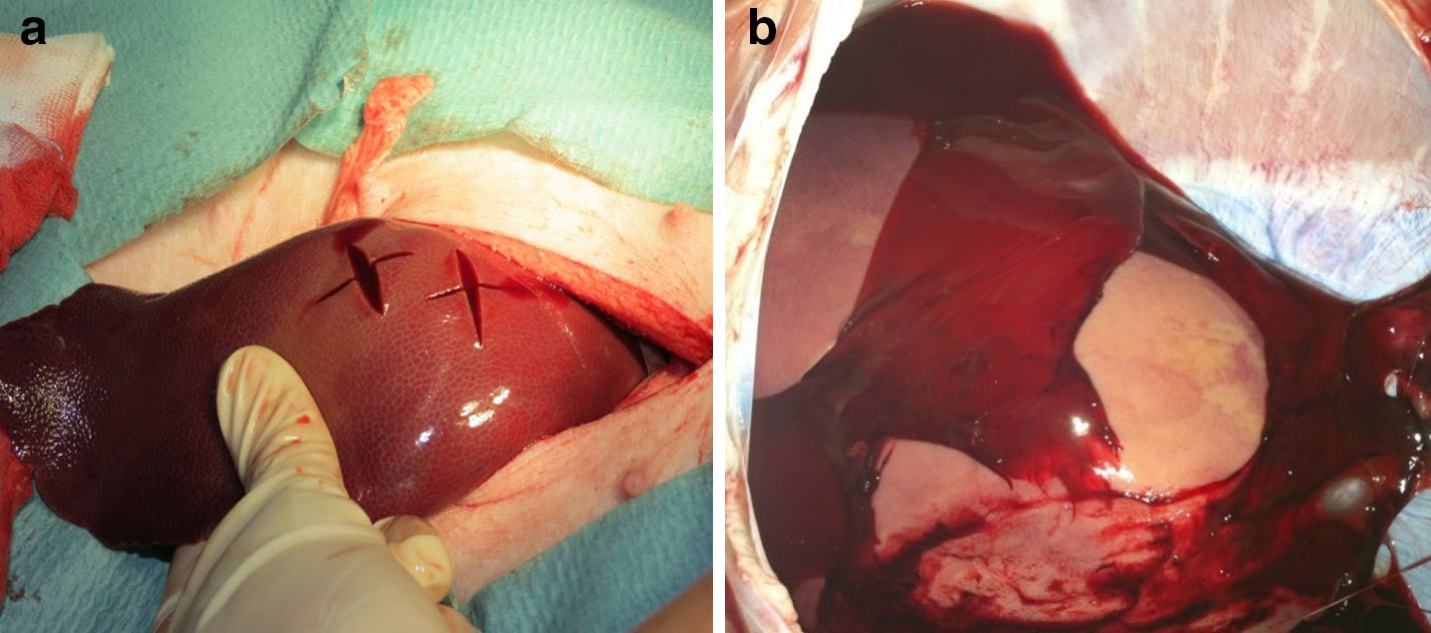
Macroscopic presentation of liver laceration. **a** Liver traumatization, **b** coagulated blood covering the liver laceration after 48 h during section

Organ-specific outcome parameters are given in Table 1. The time schedule is depicted in Fig. 1. At baseline, pCO_2_ was adjusted at 40 ± 5 mmHg and temperature was kept >37 °C. FiO_2_ was defined at 21 % during the trauma period, simulating ambient air. The animal was placed on the right side. The right hind leg was placed into a drop-weight device and 20 kg of plumb-cuboid were dropped guided from a height of 100 cm (Fig. 2). Afterwards blunt chest trauma of the right thorax (captive bolt stunner, direct contact on a panel 10 × 10 cm, upper layer of lead, lower layer of steel) was performed in the supine position, followed by laparotomy and laceration of the liver (caudal lobe) by stabbing twice with a four-edged scalpel (Fig. 3). The consequent uncontrolled bleeding was addressed after 30 s with a tamponade, consisting of seven unfolded gauze compresses. Simultaneously, systemic hemorrhage was realized by draining up to 45 % of estimated total blood volume (TBV) in group L and 50 % in group H, or until a mean arterial blood pressure of 30 ± 5 mm/Hg in group L and 25 ± 5 mm/Hg in group H was achieved. Blood loss was realized by taking repeated blood samples with a 50-mL syringe using the two-lumen hemodialysis line that was placed at the left femoral vain (two-lumen HD, 14 Fr, 15 cm, Arrow, PA, USA). We aimed to measure the blood loss in the first 20–30 min, trying to keep the MAP at the required 25–30 mmHg (with a minimum MAP of 20 mmHg). This proved to be the critical MAP for achieving cardiac arrhythmia, often with irreversible cardiac arrest in previous feasibility studies. Hemorrhagic shock was maintained for 90 min in group L and 120 min in group H.

**Table 1:** Outcome parameters

Lung	FiO_2_, pO_2_, pCO_2_, macroscopic injury, chest tubing, pO_2_/FiO_2_ ratio
Cardiovascular system	RR, HF, MAP, lactate, BE, resuscitation frequency, mortality
Liver/renal system	AST, ALT, creatinine, urinary output, AST/ALT ratio
Musculoskeletal system	CK, myoglobin, AST

Following the trauma period, fluid resuscitation was performed via warmed Ringer’s acetate solution (4 × hemorrhagic volume/60 min) to achieve physiological MAP and HR values. If needed, vasoactive drugs like noradrenaline were administered to keep MAP within physiological boundaries (55–70 mmHg).

The intervention period was followed by an observation period of 46 h under standardized intensive care management (bedding, airway and volume management, temperature control). Complications were addressed according to current standards of emergency medicine and trauma surgery, such as recommendations made in the latest update of the European Resuscitation Council [4] as well Advanced Trauma Life Support (ATLS) protocols [5]. These allow standard techniques such as insertion of chest tubes for pneumothorax. Typical emergency medications (i.e., epinephrine, noradrenaline, amiodarone, midazolame, atropine) were given intravenously as required for life-threatening events (i.e., seizure, tension pneumothorax, ventricular fibrillation, cardiac arrest).

In case of CPR, the pig was turned on its right side for external cardiac massage (chest compressions at a rate of at least 100/min, uninterrupted except for defibrillation or pulse checks). FiO_2_ was turned at 1.0, and suprarenine (1:10, 1 mg) was applied repetitively according to ERC 2010 guidelines. In case of ventricular fibrillation, amiodarone was administered (3 mL/150 mg), and external electric shocks (150 J biphasic, Corpuls3, GS Elektromed. G. Stemple GmbH, D-86916 Kaufering) were applied. Resuscitation was terminated after 45 min without achieving ROSC.

Respiratory failure (RF) was defined as more than three blood gas analyses over more than 6 h post-trauma (and after 24 h of observation) showing a pO_2_/FiO_2_ ratio <300, PEEP ≥5 in the absence of clinical or sonographic signs of severe left heart failure. Treatment was according to the ARDSnet guidelines [6].

Following the observation period, euthanasia was induced by enhancing anesthesia and applying an additional 100 µg of sufentanil, 200 mg of disoprivane, and 4 mg of pancuronium. Afterwards, 60–100 mL of potassium chloride was administered until the occurrence of cardiac arrest.

### Sample collection

We defined six points of analysis for arterial blood and urine sampling (Fig. 1): baseline, at the beginning of the experiment, immediately before the induction of trauma (1), after trauma period (2), after reperfusion (3), at 14.5 h (4), 24.5 h (5) and 48.5 h (6) post-trauma. All samples were centrifuged, (4 °C, 5 min, 4000 rpm), deep frozen and stored at −80 °C.

Euthanasia was followed by a standardized section. Tissue samples were taken from brain, lungs, heart, liver, kidneys, adrenal gland, fracture zone and ambient muscle. Half of the tissue samples were immersed in formalin 4 % and afterwards coated with paraffin wax; the other half were shock frosted in liquid nitrogen and stored at −80 °C.

### Training and preparation prior to investigation

In agreement with our animal welfare committee we obtained permission to execute a preceding feasibility study using 10 animals. The aim was to develop methods of lower limb fracture in combination with surrounding trauma, and to test the feasibility of an extended study period. This study was required for the training of participating staff and the determination of material and personal requirements. Results of this feasibility study were not included in this study.

### Statistical analysis

Statistics were compiled using Excel^®^ (Microsoft 2010) and SPSS^®^ (PASW Statistics 22). The Kolmogorov–Smirnov Test was used to test for normal distribution of data; homogeneity of variance was tested by the Levene’s Test.

Group differences were tested by the non-parametric Kruskal–Wallis Test. Then the individual groups were subjected to subsequent post hoc analysis with the Mann–Whitney *U* test, Chi-square test or Fisher’s exact test; the alpha was adjusted using the Bonferroni method to compare the intervention groups H and L and the control group. Thus, *p* < .025 was required for all tested variables concerning the three groups. Data are presented as medians and IQRs or ranges.

## Results

### Basic data

Basic data are given in Table 2. All control animals survived and none required cardiopulmonary resuscitation. In group L, CPR was needed in four of 15 animals; the return of spontaneous circulation (ROSC) was observed in two animals. The underlying cause in both cases was ventricular tachycardia leading to ventricular fibrillation and pulselessness at 24.18 and 35.5 h. Two animals died after CPR in the first 12 h of the investigation (1.48, 6.05 h) following ventricular fibrillation in the wake of uncontrolled bleeding. In group H, four animals were resuscitated, all during the intervention period; ROSC was obtained in three. Three had ventricular fibrillation, one further developed a tension pneumothorax with hypotension and asystole.

**Table 2:** Baseline data

	Group C	Group L	Group H	*p*
Number of animals	5	15	10	
Weight (kg)	35.1 (IQR 8.25)	32.1 (IQR 4.7)	36.1 (IQR6.03)	n.s.
Mortality (%)	0	13 (*N* = 2)	10 (*N* = 1)	n.s.
CPR (%)	0	26.67 (*N* = 4)	40 (*N* = 4)	n.s.
Chest tube (%)	0	40 (*N* = 6)	30 (*N* = 3)	n.s.
Hemorrhage of total blood volume (%, TBV)	–	43.8 (IQR 3,9)	49.5 (IQR 4.6)	G.C/G.L *p* ≤ 0.001 G.C/G.H *p* ≤ 0.001 G.L/G.H *p* = 0.005
min MAP (mm/Hg)	68.4 (IQR 25)	29 (IQR 9)	22.5 (IQR 5)	G.C/G.L *p* ≤ 0.001 G.C/G.H *p* ≤ 0.001 G.L/G.H *p* = 0.005
Max HR during trauma period (bpm)	95 (IQR 26)	205(IQR 28)	214 (IQR 42.5)	G.C/G.L *p* ≤ 0.001 G.C/G.H *p* ≤ 0.001 G.L/G.H *p* = 0.42
Respiratory failure (%)	0	23 (*N* = 3)	30 (*N* = 3)	G.C/G.L *p* = 0.4 G.C/G.H *p* = 0.081 G.L/G.H *p* = 0.65
Arterial lactate post-trauma (mmol/L)	1.2 (IQR 0.5)	3.2 (IQR 2.0)	3.9 (IQR 3.4)	G.C/G.L *p* ≤ 0.001 G.C/G.H *p* = 0.029 G.L/G.H *p* = 0.367
BE post-trauma (mmol/L)	6.7 (IQR 2.34)	1.5 (IQR 3.01)	1.0 (IQR)	G.C/G.L *p* ≤ 0.001 G.C/G.H *p* = 0.004 G.L/G.H *p* = 0.701
De Ritis ratio 24.5 h	0.82 (IQR 0.7)	1.78 (IQR 0.89)	3.8 (IQR 5.5)	G.C/G.L *p* ≤ 0.733 G.C/G.H *p* = 0.092 G.L/G.H *p* = 0.030
De Ritis ratio 48.5 h	1.1 (IQR 0.9)	2.06 (IQR 2.3)	3.3 (IQR 3.2)	G.C/G.L *p* ≤ 0.193 G.C/G.H *p* = 0.116 G.L/G.H *p* = 0.330

Sampled data are given as median and IQR. Group differences were tested by the non-parametric Kruskal–Wallis test. Individual groups were subjected to subsequent post hoc analysis with the Mann–Whitney *U* test/Fisher’s exact test. *p* < 0.025 was considered statistically significant

### Trauma severity

**Fig. 4:**
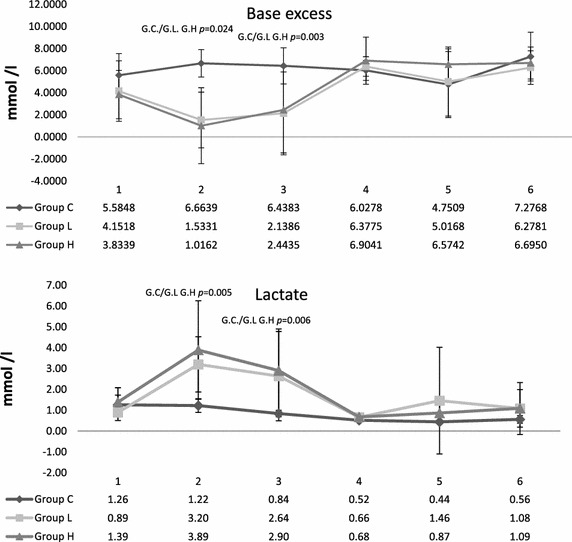
Post-traumatic arterial base excess and lactate concentrations. *1* Pre-trauma, *2* after trauma, *3* after reperfusion, *4* after 14.5 h, *5* after 24.5 h, *6* after 48.5. Sampled data are given as median and IQR. Group differences were tested by the non-parametric Kruskal–Wallis Test, individual groups were subjected to subsequent post hoc analysis with the Mann–Whitney *U* test and Fisher’s exact test. *p* < 0.025 was considered statistically significant

The severity of the trauma was confirmed by post-trauma serum lactate levels and a base deficit. In group L, lactate reached a group median of 3.2 mmol/L (IQR 2.0); 3.9 mmol/L (IQR 3.4) in group H. It was 1.2 mmol/L (IQR 0.5) (Table 2) in group C. After trauma and following resuscitation, lactate in trauma groups was significantly elevated compared to sham group (Lab. 2/3 Fig. 4). Looking at lactate between trauma groups, group H lactate was significantly greater than group L after 30 and 60 min (*p* = 0.008; *p* = 0.017), but there were no significant differences between trauma groups post-trauma. Lactate and BE were similar in all three groups after 10–12 h and over during the remaining investigation period (Fig. 4). BE was significantly less in the trauma groups than the controls during following trauma period (G.C/G.L *p* ≤ 0.001 G.C/G.H *p* = 0.004), but did not differ comparing group L and group H (G.L/G.H *p* = 0.701) (Table 2). The three animals that did not survive all had BE ≤−8 (group L −8.2 and −9, group H: −8.2 mmol/L).

### Thoracic trauma

Our blunt thoracic trauma caused visible macroscopic tissue damage (Fig. 2); pO_2_ levels did not differ during trauma (*p* = 0.119). pCO_2_ ranged 47–53 mm/Hg in group C, 47–50 mm/Hg in group L and 43–49 mm/Hg in group H.

**Fig. 5:**
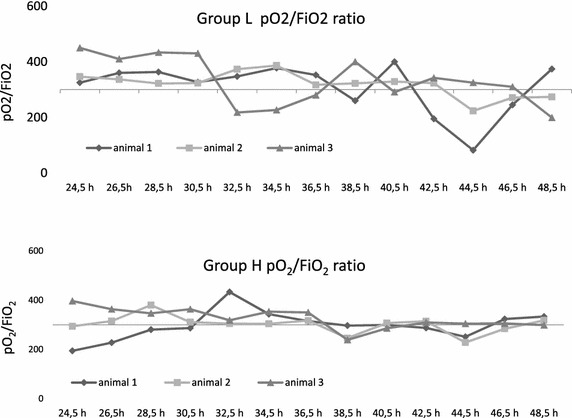
Clinical course of pO_2_/FiO_2_ ratio during the last 24 h of observation. Arterial pO_2_ was measured every 2 h by blood gas analysis (BGA, ABL700, Radiometer Copenhagen)

Three of 13 pigs in group L and three of nine in group H developed respiratory failure during the final 24 h. The course of pO_2_/FiO_2_ ratio and arterial blood gas analysis is given in Fig. 5.

Clinical suspicion of pneumothorax (subcutaneous emphysema and decreased vital capacity) necessitated chest drainage in six pigs in group L and three in group H. Differences were not statistically significant. No respiratory problems were observed in group C.

### Cardiovascular system

**Fig. 6:**
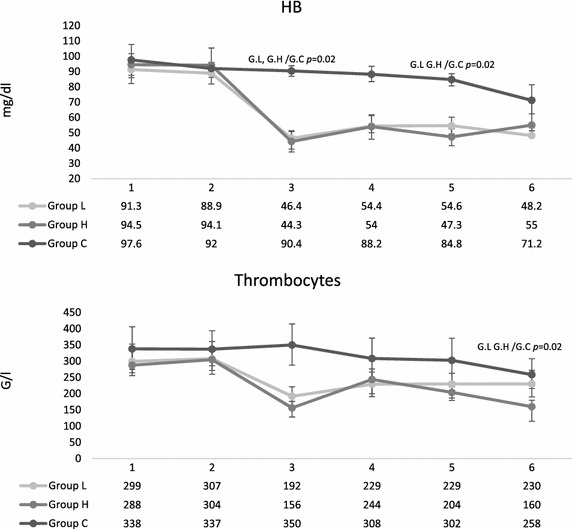
Course of hemoglobin (Hb) and thrombocyte concentrations during the experiment. *1* Pre-trauma, *2* after trauma, *3* after reperfusion, *4* after 14.5 h, *5* after 24.5 h, *6* after 48.5. Sampled data are given as median and IQR. Group differences were tested by the non-parametric Kruskal–Wallis test, individual groups were subjected to subsequent post hoc analysis with Mann–Whitney *U* test and Fisher’s exact test. *p* < 0.025 was considered statistically significant

Blood was drawn in the first 30 min of trauma. In the 15 pigs of group L, median blood loss was 43.8 % (IQR 3.9) of TBV, minimal median MAP reached 29 (IQR 9) mm/Hg and group maximum median heart rate was 205 (IQR 28) during trauma period. In group H, 49.5 % (IQR 4.6) of TBV was drawn, and the minimal median MAP was 22.5 mm/Hg (IQR 5). Group H maximum median heart rate was 214 (IQR 42.5) during shock; group C remained in the physiological range of MAP and heart rate. Heart rate was significantly increased in both trauma groups during trauma period. Following the reperfusion period, MAP reached physiological levels and was kept there during the observation period. The need for at least transient noradrenaline administration for circulatory support showed no difference between trauma groups (group L 13/15 animals, group H 7/10 animals; *P* = 0.653). None of the control animals needed circulatory support. Hemoglobin concentrations were significantly less in trauma groups after reperfusion compared to controls and remained so during the whole investigation. Thrombocyte counts also decreased significantly in the trauma groups (*p* = 0.001) after reperfusion and remained low after 48.5 h (*p* = 0.02) (Fig. 6). After 48.5 h the thrombocyte count in group H was even significantly lower than in group L (*p* = 0.013).

### Hepatorenal system

**Fig. 7:**
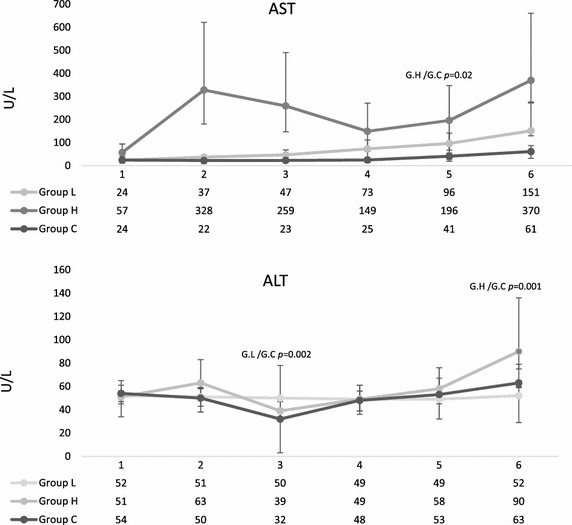
Serum AST and ALT concentrations. *1* Pre-trauma, *2* after trauma, *3* after reperfusion, *4* after 14.5 h, *5* after 24.5 h, *6* after 48.5. Sampled data are given as median and IQR. Group differences were tested by the non-parametric Kruskal–Wallis test, individual groups were subjected to subsequent post hoc analysis with the Mann–Whitney *U* test and Fishers’ exact test. *p* < 0.025 was considered statistically significant. AST norm ≤35 I/U, ALT norm ≤68 U/L

AST concentrations increased slightly over the time in all three groups, but only significantly after 24 h in group H (*p* ≤ 0.02) compared to controls (Fig. 7). There was no difference between trauma groups. The AST/ALT ratio was 1.78 (IQR 0.89) in group L and 3.8 (IQR 5.5) in group H after 24.5 h (*p* = 0.030); after 48.5 h it was 2.06 (IQR 2.3) and 3.3 (IQR 3.2) (*p* = 0.33). In six of 13 animals in group L and 5 of 7 in group H (two measurements failed because of hemolysis) AST more than tripled by the end of study period.

In one animal of group L, liver lacerations bled for the first hour after packing and required suturing and repacking. The bleeding was localized by sonographic examination following an unexplained prolonged tachycardia after the reperfusion period, which led to detection of free fluid in the abdomen (Fig. 7).

**Fig. 8:**
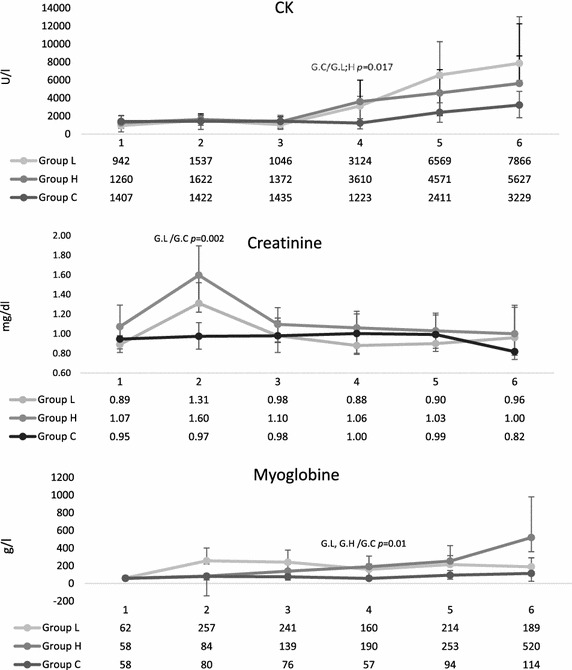
Creatine kinase (CK), creatinine and myoglobin concentrations during the observation period. *1* pre-trauma, *2* after trauma,* 3*  after reperfusion, *4* after 14.5 h, *5* after 24.5 h, *6* after 48.5. Sampled data are given as median and IQR. Group differences were tested by the non-parametric Kruskal–Wallis test, individual groups were subjected to subsequent post hoc analysis with the Mann–Whitney *U* test and Fisher’s exact test. *p* < 0.025 was considered statistically significant

As measured by creatinine clearance, renal function was significantly impaired immediately after trauma (G.C/G.L *p* = 0.002) (Fig. 8), but normalized over time and was not greater than in group C at the end of investigation. Urinary output also did not differ between groups, all groups having reduced their output after induction of anesthesia but normalized over time.

### Musculoskeletal system

All fractures were grade II according to Gustilo, Mendoza and Williams [7]. After 14.5 h serum CK was significantly increased in both trauma groups (*p* = 0.017) (Fig. 8); later it increased even in group C. Medians were >3000 U/L in group C, >5000 U/1 in group L and >7000 U/L in group H but did not differ statistically significantly at the end of investigation. Myoglobin concentrations also significantly increased in the trauma groups after the first 14.5 h. Later, in groups C and L it remained roughly constant, but group H exhibited a further, non-significant increase in myoglobin during the second 24 h.

## Discussion

We have devised a porcine polytrauma model that reflects the mortality and organ failure of polytrauma patients during shock and intensive care. We included two different severities of hemorrhagic shock. The severity of the trauma and its effects appeared to be reproducible.

Pre-clinical models are needed to develop new therapies for trauma. These are mostly porcine because of the similarity to humans [8–10]. A discussion of current models took place at a Military Medicine Workshop on Animal Models in Hemorrhage and Resuscitation Research [11], in particular the question of a reproducible trauma/hemorrhage model. The key points are as follows: (a) the need for volume- or pressure-controlled models that can allow uncontrolled bleeding, (b) surgical manipulation, e.g., soft tissue injury coincident with hemorrhage as in clinical situations, (c) significant soft tissue injury to better represent the post-injury inflammatory state, (d) a controllable degree of hypotension to lead to poor outcomes and (e) a model simulating battlefield trauma rather than one designed to meet a specific scientific goal [2, 11]. Furthermore, it was also agreed that reproducible trauma and hemorrhage are key factors and that the duration of hemorrhagic shock has to mimic the clinical situation. The pre-clinical period is 60–80 min in Germany [12].

In 2012, we presented a porcine multi-system injury model of combined chest and abdominal trauma with hemorrhagic shock, including an observation period of 15.5 h [1]. It was shown to be reproducible and fulfilled many of the above criteria. Because of the need for studies including a longer observation period under intensive care conditions [13–15], we included a fracture of the right hind leg, and extended the study period to 48.5 h under intensive care conditions. Two grades of hemorrhage were achieved during our investigation. As no previous studies used a similar time period combined with simultaneous trauma of similar severity, we gradually tested the limits of feasibility. Since we already knew, based on previous investigation that it worked at least for 15 h, we started with a hemorrhage of 45 % of total blood volume for 90 min and extended this hemorrhage to a 50 % loss of total blood volume and 120 min of shock, as described above. In addition to survival rates and resuscitation frequencies, we used lactate and BE during the trauma period as outcome parameters for trauma severity. In the early trauma period metabolic acidosis is accompanied by increased plasma lactate, reflecting inadequate tissue oxygen delivery [16]. Previous studies assessed the physiological plasma lactate concentration in Landrace/Pietrain crossbred animals at 1.1 mmol/L [17]. These correlate to the plasma lactate concentrations in group C, suggesting that the increased lactate levels in group L/H realistically represent severe trauma. There is a strong correlation between early serum lactate and mortality in trauma patients [18]. Lactate values similar to ours, ranging from about 2.5–4.0 mg/dl, were associated with a mortality of 6.4 %. Our mortality was similar, although data have to be carefully interpreted because of the inter-species differences. Both trauma groups showed significantly decreased BE during the whole trauma and reperfusion period, suggesting a significant degree of metabolic acidosis. Animals with a BE less than −8 died within 3 h. It is described, that base excess less than −6 during the first 24 h of admission after trauma is associated with a higher risk of massive transfusion, coagulopathy, ARDS and mortality [16]. Accordingly, return towards baseline values obtained by the sham group after 10–12 h was interpreted as a result of successfully performed resuscitation in trauma groups.

Oxygenation during the acute trauma period did not differ significantly between groups, perhaps because we adjusted CO_2_ levels to about 45 ± 5 mm/Hg during observation period by regulating inspiration rate and pressure.

Some independent risk factors for the development of ARDS after blunt trauma include an injury severity score (ISS) >25, the presence of pulmonary contusion, a large transfusion requirement, hypotension on admission, and age >65 years [19]. Other studies have shown specific injury patterns, such as long bone fracture, to be independently associated [20]. Post-traumatic ARDS may occur early (<48 h) or late (>48 h). Early development is also associated with presentation in hemorrhagic shock; our trauma model provides the majority of these risk factors. Three animals of each trauma group showed some degree of respiratory impairment, but no ARDS. There were no differences in lung function between groups L and H.

Porcine hemorrhage experimental models have focused on volume-controlled [18, 21, 22] or pressure-controlled [22–24] blood loss; however, both have their disadvantages. In a volume-controlled investigation, the degree of hypotension depends on many factors, at least in terms of the individual animal’s response. Most volume-controlled studies deal with a loss of 30–50 % of TBV, with a mortality rate of up to 20 % [25–28]. In most pressure-controlled studies, an MAP between 30 and 45 mmHg was generally maintained for 30–45 min. This artificial control of blood pressure does not mimic the clinical situation, and the volume of exsanguination can be very variable. Therefore, a combination of volume and pressure control may best mimic the clinical situation. Induction of a well-defined and reproducible trauma that includes significant hemorrhage is a significant factor in the establishment of a relevant trauma model. No published data deal with a withdrawal greater than 45 % of blood volume and survival beyond 24 h without retransfusion of shed blood in simple porcine hemorrhage models. Furthermore, no model of combined lung and liver hemorrhage dealing with a withdrawal greater than 45 % and survivals beyond 15.5 h is described. Models with greater blood loss did not provide observation periods longer than 6 h. In a further study, we proved that withdrawal of 45 % of TBV combined with liver laceration and lung contusion is safe and reproducible for 15.5 h [1]. Since we added a lower leg fracture and aimed to triple the investigation period, we first decided to keep the hemorrhage and the shock period similar to that of a previous study. This worked reliably for the first 15 pigs. These pigs were proven to react very sensitively, showing arrhythmia and blood loss of more than 45 % TBV or an MAP less than 25 mmHg. We slowly extended the borders of hemorrhage to 50 % TBV or an MAP 25 ± 5 mm/hg. According to cross-sectional guidelines for the transfusion of red blood cells based on clinical observation and risk factors, a hematocrit value of approximately 15 % (hemoglobin concentration 5.0–4.5 g/dL = 3.1–2.8 mmol/L) has to be assumed as the critical threshold value for RBC substitution. Blood loss greater than 50 % would have surpassed the recommended levels, indicated by the minimal Hb of 4.6 g/dL in group L. In addition, an MAP falling below 20 mmHg for some minutes was shown to promote arrhythmia and frequency of resuscitation. Therefore, we decided to extend the duration of hemorrhagic shock from 90 to 120 min to additionally aggravate ischemic injury.

As previously mentioned, animal models including withdrawal of blood until a certain endpoint for a defined period of time, followed by retransfusion of shed blood in the subsequent observation period have been described [29]. Cross-sectional recommendations for the transfusion of red blood cells define a hemoglobin concentration of 4.5–5 g/dl as the threshold value for transfusion of RBC in young and healthy subjects. Additionally, a recent publication dealing with the recommendations for red blood cell transfusion in septic patients could not prove a benefit in the transfusion of red blood cells beyond a hemoglobin value of 7 g/dl in patients experiencing septic shock. Holst and colleagues provide definitive evidence that a restrictive approach to blood transfusion not only reduced blood use by half, but also did not cause further harm to 998 critically ill patients [30]. In the presented polytrauma model, numerous factors interfere with immune response, inflammation parameters and the circulatory system. Among these are pre-existing medical conditions, iatrogenic trauma due to invasive monitoring, ventilation, and environmental factors. To improve data quality and traceability and to reduce possible interactions, we abstained from RBC transfusions. Furthermore, our pre-existing model which describes similar blood loss without severe complications due to hypovolemia, confirms our approach.

In our model this extended controlled component of bleeding and reperfusion strategy worked reliably.

Liver injury is often used to produce uncontrolled bleeding in animal models. Many combine hemorrhage and grade II–IV liver injuries, but most have a very short shock period of about 5–30 min and a maximum observation time of 7 h [31–34]. Several studies use fluid resuscitation including blood transfusion [35, 36] or surgical treatment, e.g., suturing of liver injury or the use of haemostyptic agents [37]. We decided to produce a grade II (Moore et al.) liver injury to be able to provide a 48.5-h observation period with liver trauma and impaired liver function. ALT was not elevated and there were no great differences between groups. A tripling of AST indicates severe damage; six animals of 13 in group L and 5 of 7 animals in group H showed such values at the end of study period. The AST/ALT ratio also suggests severe liver cell damage (Table 2).

However, according to creatinine clearance and urinary output, even the prolonged hemorrhagic shock in group H did not lead to clinically significant renal ischemic trauma.

We caused a proximal lower limb fracture because fractured extremities are present in more than 60 % of multiple-trauma patients [12]. This tissue trauma led to significant increases in CK and myoglobin. The differences lacked statistical significance at the late post-traumatic course, perhaps because of small group numbers and large standard deviations; shivering following anesthesia was frequent and may explain elevated CK levels in group C. The impact of muscle relaxation has not yet been evaluated.

Our study has several limitations. The animal model is limited in usefulness because of inter-species differences, and the animal welfare committee guidelines restrict sample size. Further, we were unable to achieve a great degree of post-traumatic lung injury. We thought of two ways to improve the lung injury method in advance. On the one hand, we thought of using a more widespread lung trauma at the beginning; bilateral hits were discussed, but this idea was ignored because we wished to have an uninjured lung for data comparison. On the other hand, we tried an aggravation of force by reducing the upper layer of plumb and using a more powerful munition for the captive bolt stunner. Following this, we observed a great amount of tension pneumothoraxes and cardiac arrhythmias due to concomitant heart contusions, resulting in cardiac arrest. Beyond this, a combined repetitive surfactant washout could be promising, but it has to be considered that it would provide a kind of artificial (and not trauma-induced) lung injury. According to the National Trauma Databank, incidence of trauma-related ARDS in trauma patients requiring mechanical ventilation for greater than 48 h is 6.5 %. Hence, to achieve a sufficiently large number of ARDS cases, increase not only in the observation period but also in the number of animals is needed.

As a further limitation, we conclude that any ischemic renal injury was too short to impair the outcome of our study, although changes in renal function may become significant later in the post-traumatic course. Additionally, we have taken into account that creatinine measurement is perhaps not appropriate for detecting early hypoxic-induced renal failure, with the consequence that more sensitive biomarkers for renal injury, such as TIMP-2, IGFBP-7 cystatin C, and NGAL have to be considered in further investigation.

Furthermore, we decided to use Ringer’s acetate solution for resuscitation. Some trials show that there is evidence that balanced salt solutions are beneficial towards NS as a means of preventing development of hyperchloremic metabolic acidosis. Until now there is no high-quality randomized controlled trial comparing different balanced crystalloids to each other and, therefore, no consensus exists on a single preferred solution [38, 39]. Nevertheless, even balanced Ringer’s acetate solution contains some chloride that can induce a metabolic acidosis.

At last, the trauma groups were too close to each other (in terms of blood loss and duration of shock period) to achieve significant differences. Nevertheless, a higher blood loss of about 60 % with a comparable shock period led to a need for vasopressors and a higher frequency of CPR, which led to a high mortality in previous feasibility tests. As we aimed to obtain 46 h of observation following trauma, a balance between mortality and reaching a sufficient severity of trauma was hard to find; this was also the case because of differences in shock tolerance between individual animals.

Last but not least it can be assumed that a further extension of the observation period may have revealed greater severity of trauma.

## Conclusion

Our model of polytrauma appears to reflect the mortality and organ failure of polytrauma patients during shock and the intensive care period. It was possible to manage fluids, ventilation and complications according to our goals. Further aggravation of hemorrhagic shock led to increased organ injury and resuscitation rates, thus the degree of trauma must be chosen to reflect the aims of the investigation. This experimental protocol could be of use for the assessment of therapeutic approaches during the post-traumatic period.

## Abbreviations

ATLS: Advanced Trauma Life Support
BE: base excess
CPR: cardiopulmonary resuscitation
I:E: inspiration-to-expiration ratio
IGFBP-7: insulin-like growth factor-binding protein 7
ISS: injury severity score
MAP: mean arterial pressure
NGAL: neutrophil gelatinase-associated lipocalin
PEEP: positive end-expiratory pressure
PiCCO: pulse contour cardiac output
RBC: red blood cells
RF: respiratory failure
ROSC: return of spontaneous circulation
TBV: total blood volume
TIMP-2: tissue inhibitor of metalloproteinases 2
